# Use of a scattered light sensor for monitoring the dispersed surface in multimodal system

**DOI:** 10.1007/s00216-025-06038-0

**Published:** 2025-08-02

**Authors:** Lukas Schmitt, Stephan Scholl, Matthias Rädle

**Affiliations:** 1 CeMOS - Research and Transfer Center, Technische Hochschule Mannheim, Paul-Wittsack-Straße 10, 68163 Mannheim, Germany; 2 Institut für Chemische und Thermische Verfahrenstechnik, Technische Universität Braunschweig, Langer Kamp 7, 38106 Brunswick, Germany

**Keywords:** Backscatter sensors, Crystallization monitoring, Dispersed phase, Optical spectroscopy, Process control, Multimodal particle size distributions, Mie scattering

## Abstract

The article presented here deals with the quality parameter dispersed surface in material systems like suspensions or dispersions in which several phases are present simultaneously such as solids in continuous solution. Here, other quality parameters are relevant for the quality of the products than, for example, in pure solutions or gases. The dispersed surface is relevant for reaction rates, absorption capacity, and many other properties of the suspension. In contrast to the common method of measuring particle size distributions and solid concentrations in a complex manner, the article shows that the use of a simple scattered light measurement is also sufficient for disperse phase measurement. Based on previous investigations with monodisperse material systems “Use of a scattered light sensor for monitoring the dispersed surface in crystallization” (Schmitt et al., *Chem. Ing. Tech.*, Bd. 94, Nr. 8, S. 1177–1184, 2022), the optical effect of mixtures of fine and coarse particles is discussed here. It is shown that the dispersed surface can also be determined directly in materials characterized by very broad particle size distributions using scattered light probes. In addition to the pure measurement results, the description of the measurement setup and derived findings, the article deals with several side aspects of disperse-phase systems. The specific dispersed surface of the dispersed phase in suspensions, emulsions, bubble columns, and aerosols plays a decisive role in increasing heat and mass transfer processes. This has a direct impact on the space–time yield in large-scale chemical process engineering production plants. The results obtained contribute to a comprehensive understanding of the dispersed surface and enable improved control and optimization strategies in the chemical engineering industry.

**Graphical Abstract:**

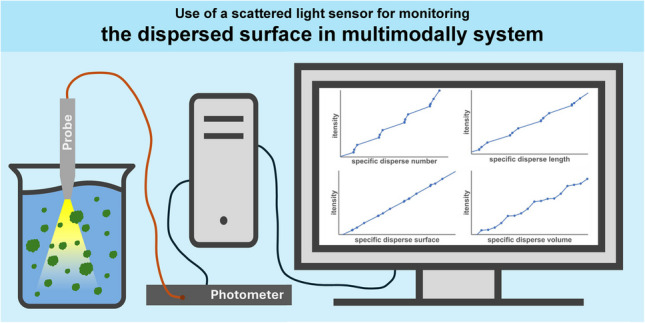

## Introduction

Disperse systems are a widely used product form in the field of chemical, thermal, and pharmaceutical process engineering. Disperse systems are mixtures of substances in which several different phases are present. These can be particulate solids dispersed in solutions. However, the term disperse system also includes emulsions—i.e., droplets of substances in a continuous liquid phase—aerosols, i.e., particles in gases or air, and more complex systems such as suspension emulsions, i.e., particles and droplets in a continuous liquid phase. In the following, we use the term disperse surface to denote the total interfacial area between the dispersed particles and the surrounding continuous phase. This parameter differs from the general term disperse phase, which refers to the particulate or droplet phase present in the system, regardless of surface properties. These mixtures of substances occur in the process engineering units crystallization, precipitation, emulsification, drying, comminution, agglomeration, and many others [2–4].

The aim of this article is to present a measurement technique that enables an improved interpretation of suspensions, i.e., particles in liquids. We will therefore focus on suspensions in the following. Suspensions are sometimes also referred to as dispersions in the literature. The boundary is floating. Small particulate systems with, for example, nanoparticles up to a particle size of a few micrometers are often referred to as dispersions and particulate systems above the micrometer limit up to millimeters as suspensions [5–7]. Depending on the application, particles occur in different size ranges. In the wet comminution sector, for example, for obtaining pigment pastes or paints, the particles are often well below 1 μm in particle size [8].

In the area of crystallization, the particles are often larger than 1 μm up to several hundred micrometers or even millimeters [9, 10]. Depending on the size range, different monitoring instruments have been established. Overall, the monitoring and quality and process control of particulate systems/disperse systems, such as suspensions in our case, are much more difficult than the monitoring of solvents [11, 12]. Near-infrared spectroscopy has become widely used for solvents. This is because common solvents give off a kind of fingerprint [13–15]. Furthermore, it is often possible to transilluminate a solution, which means that a measurement path can be realized in transmission, for example in bypass, in a transmission cell or as an inline transmission probe. For higher solution concentrations, such as in the production of dyes for the textile industry, ATR technology has proven its worth [16–22].

This measurement technique, known as attenuated total reflection, uses a quantum mechanical effect to scan a very thin boundary layer at the interface between the measuring device and the solution. This makes it possible to limit this boundary layer to a few micrometers without having to construct an actual flow cell. If it is possible to achieve an optical density of 3 extinctions per 3 μm, for example, with the usual measuring ranges of spectrometers in the range up to 3 absorbances, this would correspond to 1000 absorbances per millimeter of transmission distance. It can be seen that such high optical densities at these concentrations cannot be monitored in transmission, which is why ATR technology has become established. Apart from its use with dyes in the visible range, ATR spectroscopy in the mid-infrared region has also become widely established for the analysis of other solutions [23–31].

The longer wavelengths also increase the penetration depth to a range of 10 to 30 μm, which leads to reasonable extinctions for common solvents that fit well into the sensitivity range of current measuring devices. Turbidity is now typically the range where particles can also be found in the solution. In practical applications, this leads relatively quickly to the fact that normal transmission arrangements can no longer be used, as the usual turbidity of precipitating particles, for example, precipitation reactions or crystallization, is so high that a receiver in a transmission device can no longer detect anything meaningful [32]. It is important to realize that an extinction of 4, which is typical when monitoring the quality of black beer for its color, means that only one ten-thousandth of the light passes through the solution. This value refers to a standard optical path length of 10 mm, as specified in MEBAK guidelines and formalized in DIN 8777 [33]. For this reason, classic turbidity meters that operate using a transmission setup are not suitable for monitoring technical suspensions. These devices are suitable primarily for monitoring low turbidity levels, such as in filter applications where turbidity is minimal or when a simple yes/no indication is needed to determine filter integrity and detect cracks.

A different technique is therefore required for the normal technical processes of crystallization, precipitation, sedimentation, or phase separation processes. This is where a scattered light measurement technique based on backscattering comes into play [34–42]. In this article, we will show and demonstrate using very simple system examples that high concentrations of particulate loadings of up to 10% by weight can be monitored using this technology. However, it should be noted that backscattering also involves challenges that should not be overlooked when compared to transmission measurement. The measurements and results presented in this article demonstrate that the usual signals of common scattered light probes do not directly correlate with particle concentration. Rather, it will be shown that they are associated with the specific surface area of the particles. This contradicts the usual information provided by the device manufacturers, which specify concentration ranges and output concentrations. However, it should be noted that this is only justified when calibrated to the corresponding material.

If the material or one of its properties changed, the characteristic curve that relates the sensor signal to the dispersed surface shifts and the device must be recalibrated. In addition to the material composition, an important property of particulate systems is the particle size distribution and the reflectance property of the surface, which is usually a spectral function. However, the signal that is the focus of this article, the specific surface area, is independent of the particle size.

### Process control in disperse-phase system

One way of determining the dispersed surface is the online determination of the particle size distribution (PSD) and, separately, the concentration. Laser diffraction is very often used for PSD measurements. This method is very complex due to the required sampling (e.g., bypass) and dilution of the suspension in a saturated solution, such as in crystallization processes or suspensions in solvent-saturated solutions, and is therefore rarely performed. Another established method is the high temporal resolution focused beam reflectance measurement (FBRM), which works with rotating lasers and can be used at high concentrations [43, 44]. This method requires a great deal of equipment and, compared to the proposed simple method based on scattered light, can only be used to a limited extent in potentially explosive environments. An emerging method relies on image analysis, which is currently not applicable at high concentrations, but will be possible in the future by using methods based on artificial intelligence [35].

Optical turbidity measurement based on transmission requires dilution of suspensions to obtain a measurable signal, which under ideal conditions could relate to dispersed surface. This need for dilution makes inline monitoring in technical processes such as crystallization, which is a particularly important application, impractical and limits the applicability of transmission measurements in these environments. In this work, miniaturized inline sensors with a high sampling frequency and low measurement volume in backscattering are used to solve the problem [1, 45]. It is shown that this works surprisingly well over a wide concentration range and shows no pronounced Lambert–Beer behavior, i.e., the intensity does not decrease with increasing concentration, which is typical for transmission. This results, as will be shown, from the design and miniaturization.

**Fig. 1:**
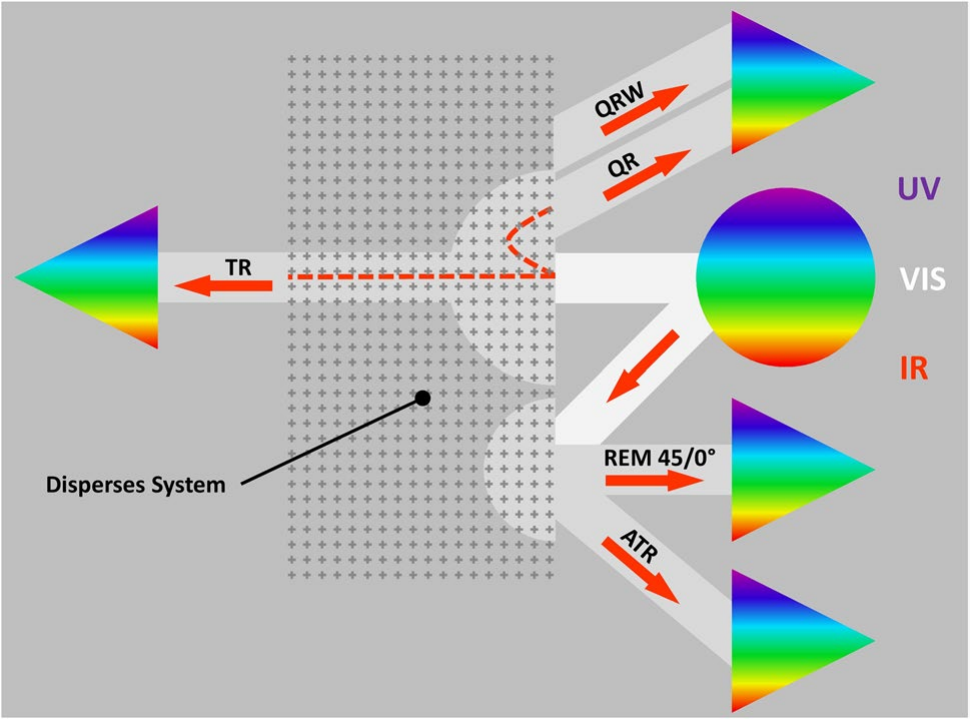
Principle possibilities of suspension tracking with optical methods

The main geometric arrangements commonly used in optical suspension monitoring are illustrated in Fig. 1. Here ATR means attenuated reflection, REM remission, QR quasi backscattering, QRW quasi backscattering with wide spacing, TR transmission. Other configurations such as detection at 90°, illumination of larger areas, or insertion of optical fibers into the suspension are possible but not depicted here for clarity.

**Fig. 2:**
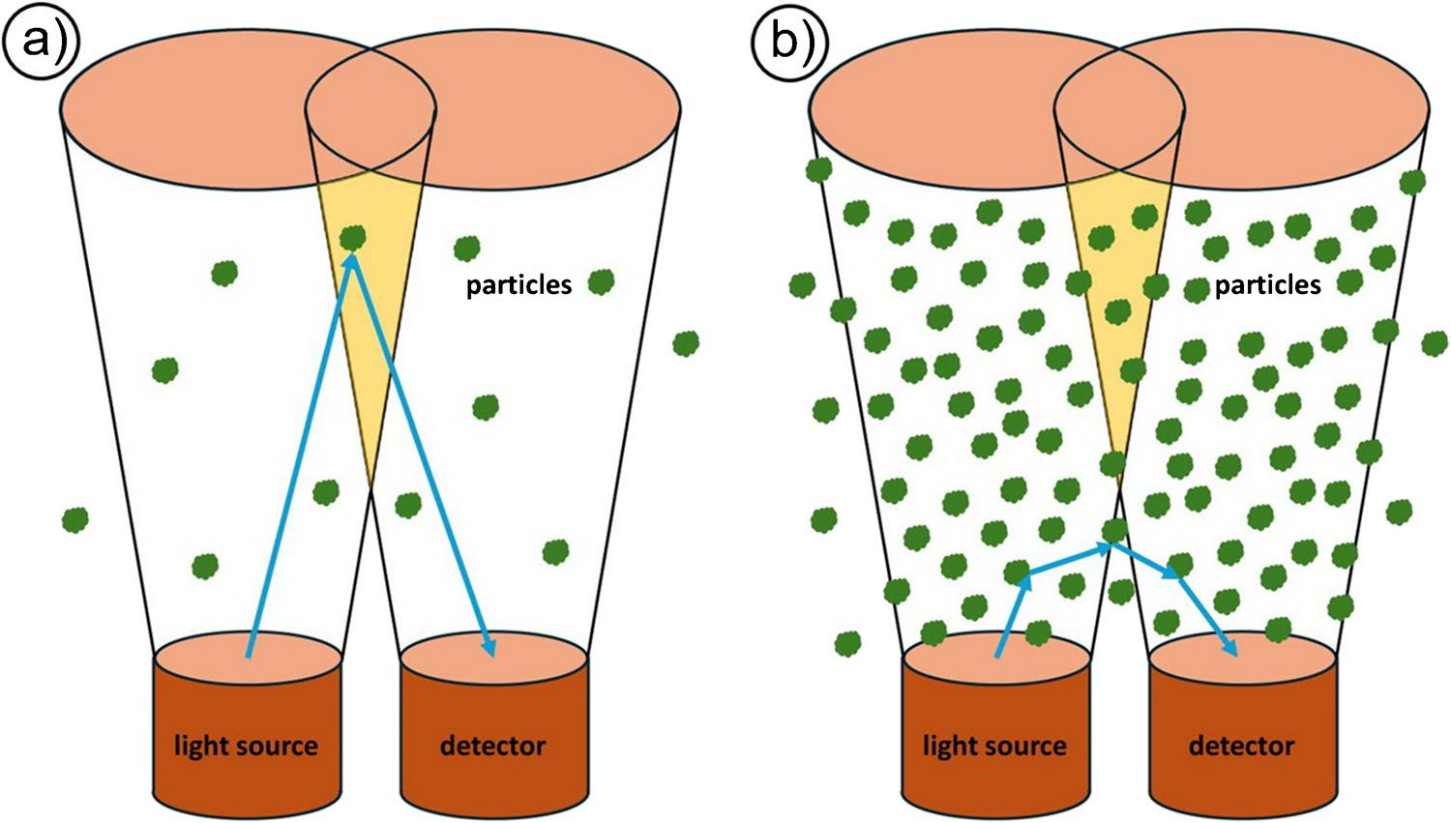
Principle of quasi backscattering, light source and receiver are offset

When the particle concentration in a suspension is low, the light emitted by a light source is predominantly scattered by individual particles and returns to the detector as a remitted signal after being scattered once (Fig. 2a). With increasing particle concentration, however, the probability increases that the light is scattered several times by different particles before it reaches the receiver. In this case, we speak of multiple scattering (Fig. 2b). The number of these scattering processes increases with the concentration of the disperse phase. The spatial distribution of the scattered light intensity depends largely on the ratio of the particle diameter to the light wavelength used. The more frequently the light is scattered on its way to the detector, the more the characteristic directional distribution changes. With increasing particle concentration, this results in a gradual alteration of the reflectance properties across different wavelengths. As the angular dependence of light scattering can be physically described by models such as Fraunhofer diffraction and Mie theory, a dependence of the reflectance behavior on the particle size is to be expected.

In suspensions, the particle shape is of particular importance. It has an effect on the scattered light distribution. The typical phenomena are represented by scattering, diffraction, refraction, and absorption. For spherical particles and low concentrations, the quantities can be represented using Mie theory [46–49]. This theoretical framework enables the computation of angle-resolved intensity distributions based on particle size, refractive index, and wavelength. Free program versions, e.g., from Philip Laven, allow simple calculation of the distributions. Within particle size ranges relevant to applications such as paint and coatings, Mie theory predicts a relatively flat angular dependence in the backscattering regime (160°–180°) for certain size regimes, facilitating a more robust interpretation of measured intensity signals in this angular domain.

In the article presented here, a technique will be used that combines the possibility of use at high concentrations with high robustness in a direct production environment. This technique, described by Polke et al. [32] as quasi backscattering technology, often uses fiber optic technology so that the light source and receiver visible to the substance are both fiber optic ends. These can have diameters of 100–1000 µm and can therefore be easily integrated into the respective process environment.

**Fig. 3:**
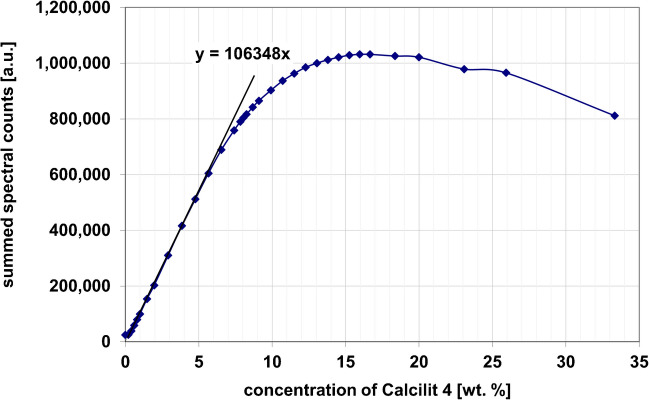
Scattered light signal as a function of particle concentration for quasi backscatter sensors in Calcilit suspension with D50 of 4 µm. Figure adapted from [34], with modifications and emphasis added by the authors

Due to the miniaturized design, the incident light only has to penetrate a few tens to hundreds of micrometers of suspension. The technology is therefore suitable for process control in the presence of high concentrations. In Fig. 3, a suspension of limestone Calcilit G4 in water is measured as a test case. Calcilit G4 is a finely classified calcium carbonate with a defined particle size distribution. A reflectance probe was used to measure the backscattered light from the suspension. The Calcilit concentration was varied between 0 wt% and 33 wt% [34]. For the evaluation, a spectrometer was used. The total spectral counts over the detected wavelength range were summed and plotted as a function of the Calcilit concentration. The signal initially increases linearly and then saturates and drops, which, according to Guffart et al. [35] is due to absorption processes that eventually overcompensate for the increasing scattering. Different technologies can be used as detectors. For example, spectrometers can also be connected to the receiving optical fiber.

**Fig. 4:**
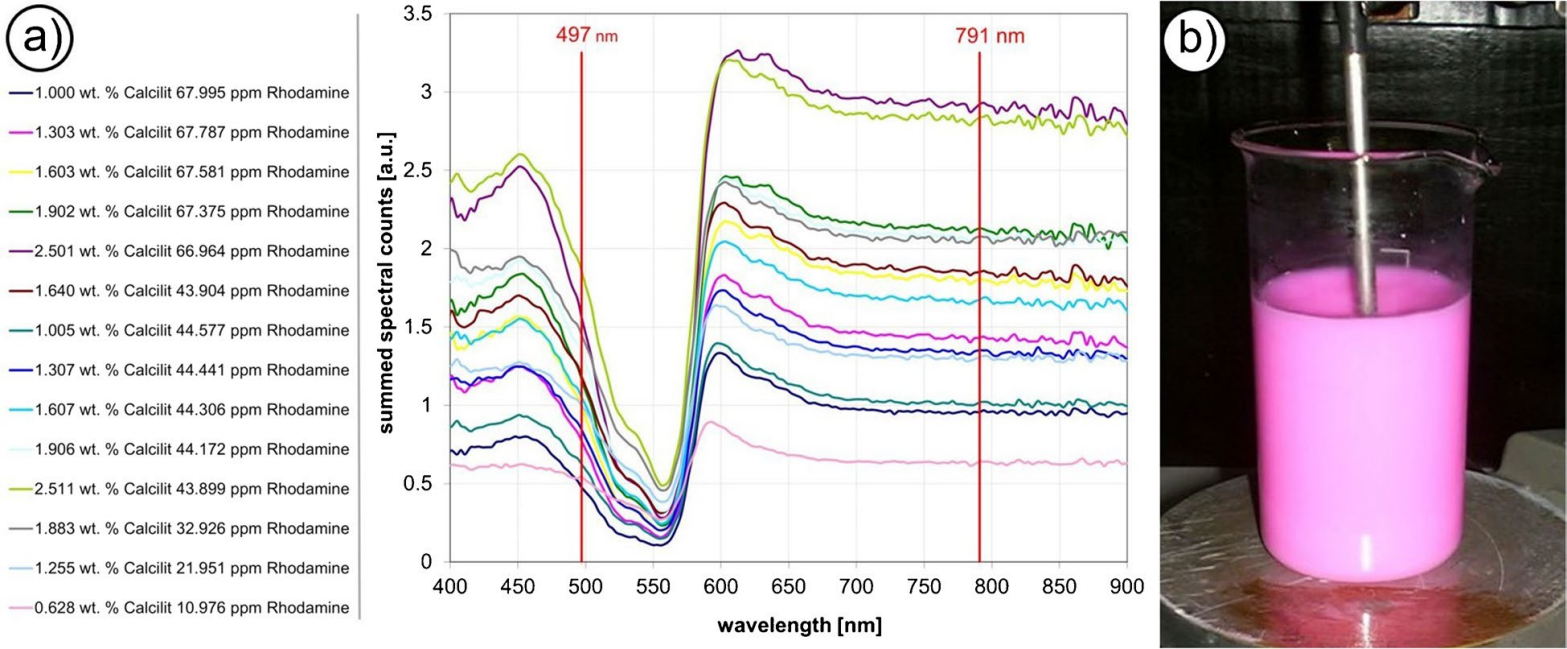
Mixture of highly concentrated Calcilit suspension and Rhodamine base B; **a** signal progression; **b** experimental illustration. Figure adapted from [34], with modifications and emphasis added by the authors

Wavelength-selective detection can be useful for distinguishing different components in turbid media. As illustrated in Fig. 4a, remission at certain wavelengths (e.g., 791 nm) correlates predominantly with the particle concentration, while other wavelengths (e.g., 497 nm) are more strongly influenced by the absorption properties of dissolved substances such as dyes. A total of 14 suspensions were prepared consisting of water, Calcilit G4, and Rhodamine as a model absorber. Two measurement series were conducted in which the Rhodamine concentration was held constant while the Calcilit concentration was systematically varied. Additionally, three further suspensions were examined, each with varying concentrations of both Rhodamine and Calcilit, in order to investigate the combined effects of absorption and scattering on the optical response. This enables an indirect separation of the particle-related and solution-related contributions to the overall remission signal.

**Fig. 5:**
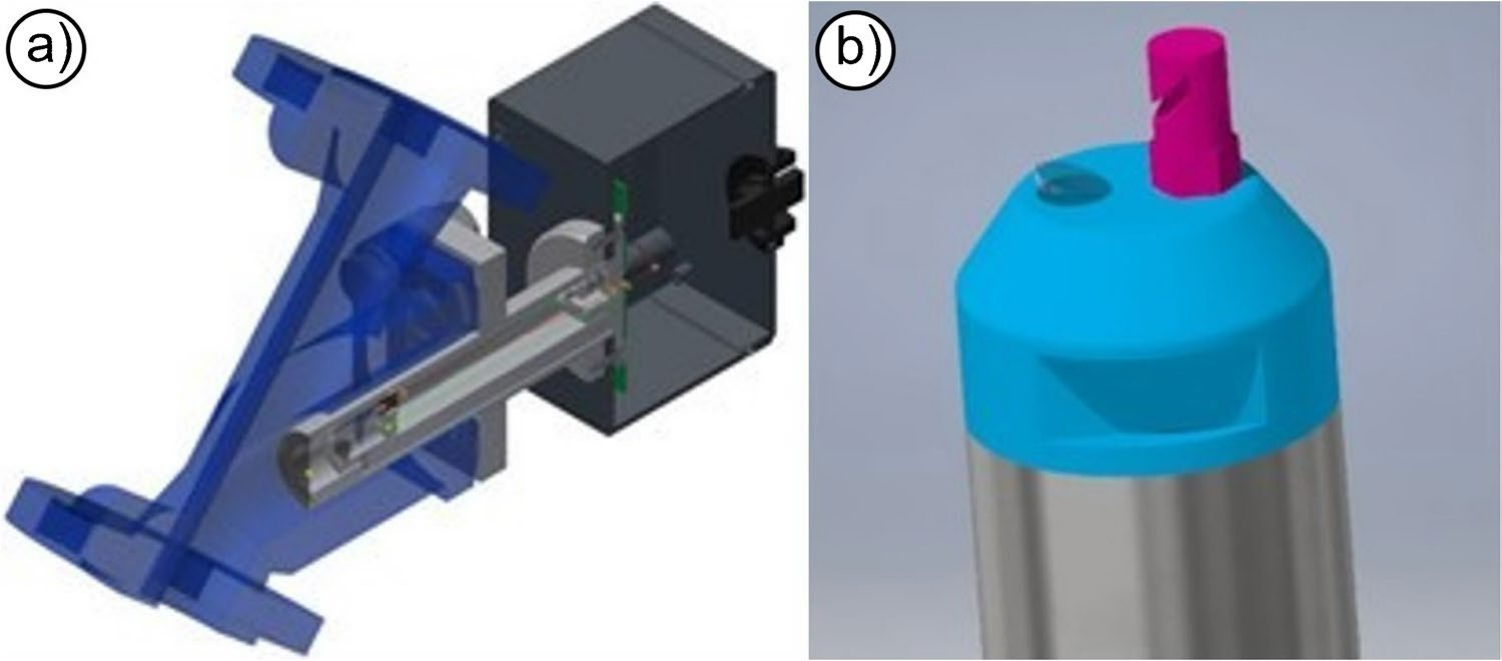
Fiber optic sensors **a** installation option in pipelines; **b** probe design with a high-pressure jet cleaning

In an industrial environment, such fiber optic sensors can be installed in different ways and in adapted probe design. Figure 5 illustrates two practical implementations of fiber-optic probes in industrial environments. The sensor shown in Fig. 5a is based on the backscattering principle and is embedded in a custom housing designed for direct installation into a pipeline system. This allows for continuous inline monitoring of turbid media. Figure 5b depicts a rod-shaped probe with a flattened optical window at its tip. Behind this window, optical fibers for both light source and signal collection are embedded. To ensure long-term functionality in harsh or fouling environments, the probe is equipped with an integrated high-pressure cleaning nozzle (purple component), which periodically cleans the optical window.

## Material and methods

### Simulationsprogramm

In order to understand the results found, a program written by Philip Laven was used, which is based on Mie theory and provides calculations of the expected scattered light intensity. The problem with scattering from particles is that the angular distribution of the scattered and diffracted light changes with the particle size [46–49]. Larger particles generally scatter more strongly in the forward direction, while smaller particles tend to scatter isotropically [50]. The sensor used measures the scattering of the light in approximately 180° backward scattering. Due to the special design of the sensor, the 180° scattering is not used directly. To increase the robustness of the sensor, the transmitting and receiving fibers are positioned next to each other. This avoids the Tyndall effect, which in unfavorable cases leads to light deposits or scratches on the sensor surface resulting in a strongly superimposed scattered light signal, which does not come from the substance but from the sensor itself [51–53].

This situation can be avoided if the light source and detector are next to each other. Slight impurities on the emitter fiber or slight damage such as scratches, deposited crystals, dirt particles, etc. reduce the irradiation of the light into the suspension, but the light scattering caused by the impurities does not reach the detector. This arrangement therefore achieves scattering angle ranges of approx. 170 to 175°. The theoretical consideration is now to see whether the backscattering changes significantly in this angular range with regard to the calculation methods for individual particles. As we know from the previous paper, the backscattering signal changes with the dispersed surface for monodisperse particles [1]. We expect problems if the result of Mie scattering in the form of the extinction coefficient or, in particular, the backscattering coefficient depends strongly on the particle size in the angular range mentioned. In the following simulations, the integral of the scattering from 170 to 175° is shown over the particle size in the investigated range. The investigated range goes from approx. 2 µm particle size to approx. 100 µm particle size. If the mixture is generated, it is no longer possible to tell from the signal whether the scattering is caused by large or small particles [50].

### Electronics

The photometer is used for the precise detection of optical signals in application-specific measurement scenarios. It combines modern components of detection, excitation, temperature control, and control technology to ensure high dynamics, stability, and user-friendliness. The design, functional principles, and technical features of the four main components are described in detail below.

#### Detectionmodul

The detection part is responsible for the precise conversion of incoming light signals into digital data. Silicon PIN photodiodes [54] are used as sensor elements, which were selected due to their high quantum efficiency in the visible to near-infrared spectrum (400–1100 nm) and their low dark current noise characteristics [55]. The generated photocurrent is processed by the highly integrated AS89010 current-to-digital converter [55]. This IC enables a parameterizable integration of the input signal over a period of 1 ms to 1024 ms, which considerably extends the dynamic range of the system [54]. In addition, the sensitivity range can be adjusted in 16 steps from 20 fA/LSB to 5000 pA/LSB to capture both weak and intense light signals without saturation effects. The converter uses a delta-sigma method with an integrated low-pass filter to suppress high-frequency interference (e.g. mains hum) and periodic noise. Once the measurement is complete, the result is available as a 16-bit data word in the internal register. A dedicated interrupt pin (INT) signals to the controller that the data is ready, which is then read out via the I^2^C bus (operating frequency: 400 kHz) [56]. The combination of analog signal integration and digital filtering reduces the calibration effort and increases the reproducibility of the measurements.

#### Excitation unit

Power-optimized LEDs [57], which emit in the wavelength range of 450–650 nm [57], are used as light sources. To stabilize the luminous flux, a precise constant current source (KSQ) is implemented with the LT3092 linear regulator [58]. The LT3092 enables output currents of up to 200 mA with a control accuracy of ± 1% and a low noise level (< 35 µVrms) [58]. The operating current of the LED is initially set roughly via solder bridges (selection range: 20 mA, 25 mA, 30 mA, 40 mA, 50 mA, 60 mA, 70 mA, 100 mA) and then fine-adjusted via a multi-turn spindle potentiometer (10 turns, ± 1% linearity). Control is via an ENABLE input, which enables the LEDs to be activated in sync with the detection cycle. This minimizes thermal drift effects due to continuous operation and increases the service life of the LEDs [43, 44]. The temperature control is monitored by a platinum resistance temperature sensor (RTD, KS-PT100A-3.0–330-4L,) with an accuracy of ± 0.1 °C in the range from − 50 °C to + 300 °C [59]. The RTD is read out via the MAX31865 amplifier module (Adafruit Industries LLC, USA), which offers a 15-bit resolution and compensates for resistance deviations using a precise reference resistor (4300 Ω, ± 0.1%) [58]. The IC implements a 4-wire compensation circuit to eliminate lead resistance influences. Communication takes place via SPI, whereby the controller receives raw data (16-bit resistance ratio) and converts it into temperature values using a Callendar-Van Dusen equation implemented in software [45]. An integrated error detection system reports interruptions or short circuits in the RTD sensor.

### Fiber optics

The fiber-optic system is used for the connection between the electronic measuring unit and the sensor head. It is based on a backscattering principle in which the transmitting and receiving fibers are arranged in close proximity to each other. This configuration minimizes direct coupling losses between the fibers and ensures high signal quality. The light emitted by the transmitting fiber interacts with the sample matrix, whereby part of the backscattered radiation is captured by the receiving fiber and directed to the detector. Due to the limited expansion of the light paths in the range of a few millimeters, the measurement intensity remains closely coupled to the specific surface structure of the crystal material. This enables precise measurements even with high crystal contents.

The physical construction of the probe follows a compact design with a length of 50 cm and a diameter of 10 mm. The tip of the probe is conical to optimize the flow behavior. A platinum resistance temperature sensor is installed in the probe tube at the probe tip close to the measuring point. The optical fibers used have a core diameter of 400 µm and a total length of 3 m. The arrangement of the fibers in the probe tip comprises three tightly bundled fibers and a separately positioned single phase. This configuration allows both the targeted detection of backscattered signals and the detection of extraneous light for calibration or interference signal reduction.

For the interface connection, the optical fibers are coupled to the measuring device via standardized SMA connectors, which ensures stable and low-loss signal transmission. For increased operational reliability, two additional fibers were implemented as redundancy to compensate for potential fiber breaks or to enable alternative measurement configurations in future experiments. The isolated single phase can also be used to investigate the coarse particle size distribution, allowing further analysis of the sample structure in subsequent studies. The same fiber-optic system was used as in the paper 10.1002/cite.202200076 Schmitt et al. [1].

### Laboratory crystallizer

**Fig. 6:**
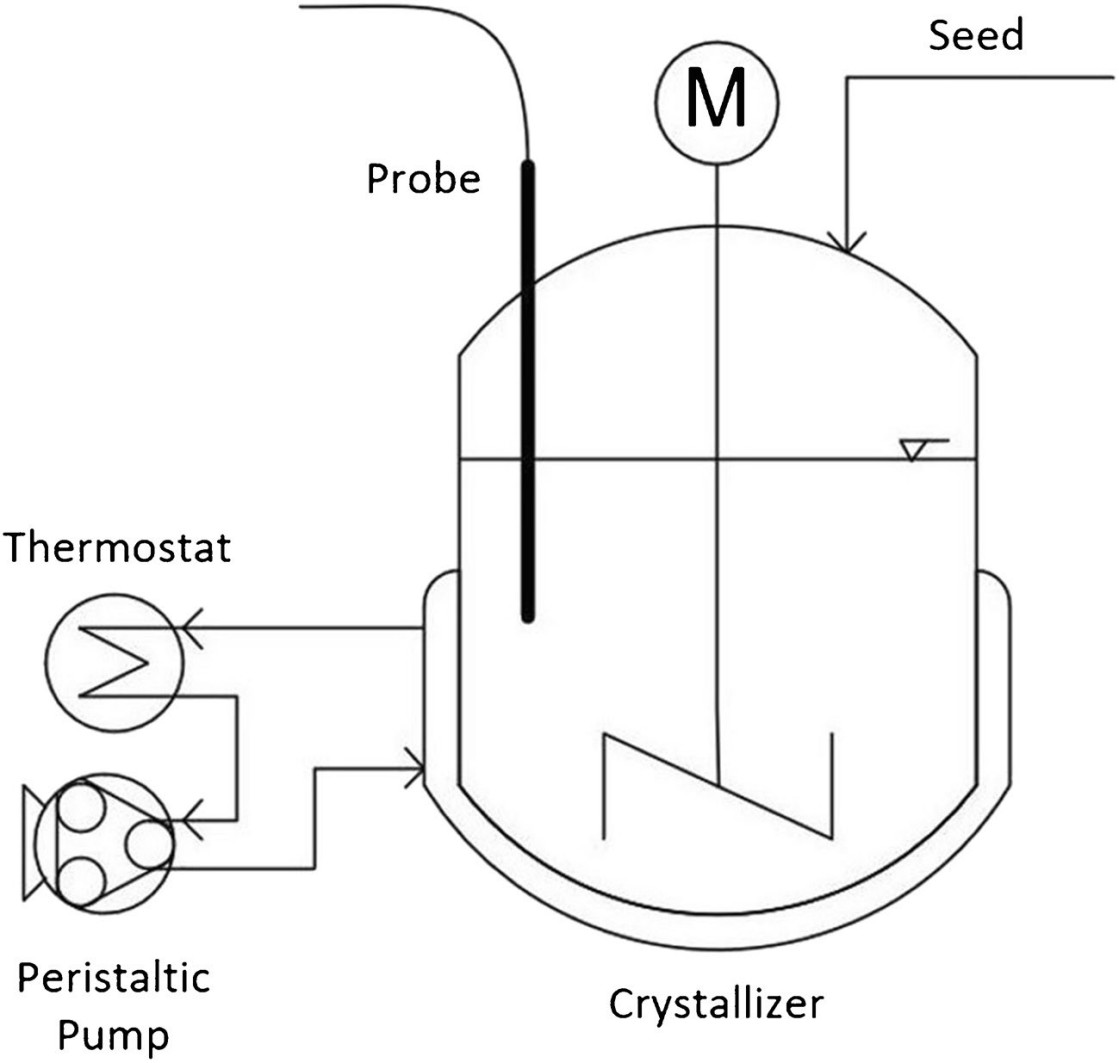
Laboratory crystallizer [1]

The experiments were carried out in a laboratory crystallizer (see Fig. 6). A double-walled 2-L glass vessel was used as the stirring vessel. The temperature of the glass vessel was controlled using a Huber thermostat (Ministat), which was coupled to a peristaltic pump (Ismatec VP). The suspension was mixed using an RW 28 basic laboratory stirrer (IKA), which was equipped with a three-blade turbine stirrer with a diameter of 70 mm. A black elastomer mat was glued in place to avoid light reflections on the floor.

The sensor was positioned to ensure that its tip did not face the stirrer, preventing signal distortions caused by reflections from the metal surface. To shield it from extraneous light, the entire stirring vessel was covered with a black cloth. Insoluble particles of the test substrate were introduced through a funnel in the vessel lid.

### Test substrate

Glass beads from Sovitec were used as the test substrate for the measurements. These were the four products Microperl, Omicron NP5, Omicron NP3, and Starmixx. The particle size distributions of the glass beads used were determined using a laser diffraction-based particle measuring device (Sympatec HELOS). The Sauter diameter d23 of the respective products was used as the equivalent diameter for evaluating the measurements. This was 2.093 mm for Omicron NP3, 4.089 mm for Omicron NP5, 6.604 mm for Microperl, and 99.149 mm for Starmixx.

## Experimental investigations

To quantify the dependence of the scattered light signal on various parameters of the particles, four types of glass beads (Omicron NP3, Omicron NP5, Microperl, and Starmixx from Bassermann) with a defined size and narrow particle size distribution were dispersed in 1.5 kg of water and measured under stirring. A stirrer speed was selected that prevented the glass beads from settling on the bottom of the laboratory crystallizer, but no air was stirred into the water.

At the beginning of the measurement, the offset signals were recorded without glass beads. Glass beads were then added in 2.5 g increments up to a total mass of 200 g. The glass beads were added in the following order: Microperls, Omicron NP3, Omicron NP5, and Starmixx. After each addition, dispersion was carried out for 5 min, and the signal strength was continuously recorded. The data acquisition rate was 64 ms. The suspension was tempered to 20 °C during the measurement. The scattered light value without spheres was subtracted as a zero signal in each case. This was achieved at a speed of 300 rpm.

## Results and discussion

**Fig. 7:**
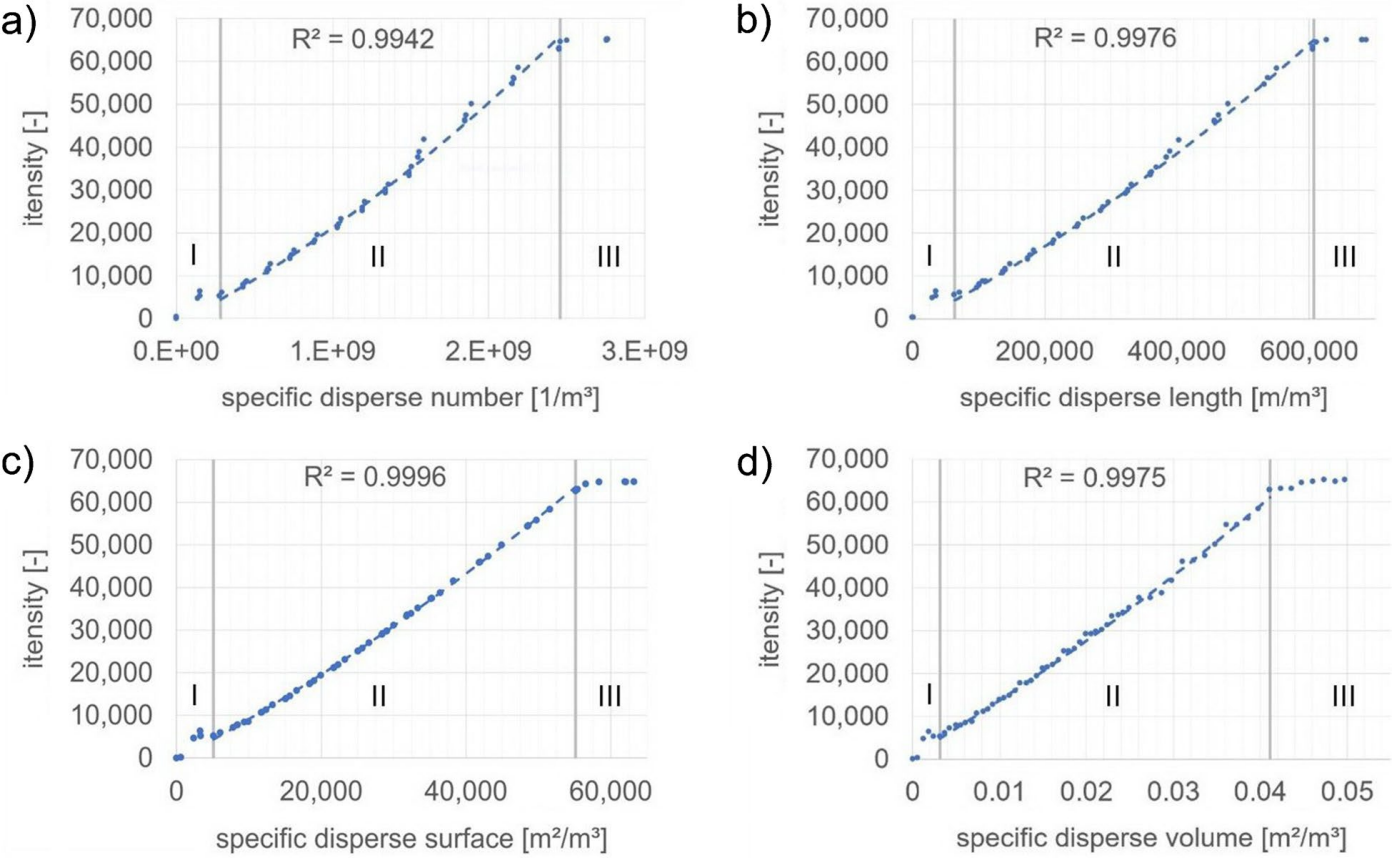
Sensor signal of dispersed glass beads over **a** specific dispersed number; **b** specific dispersed length; **c** specific dispersed surface; **d** specific dispersed volume

**Fig. 8:**
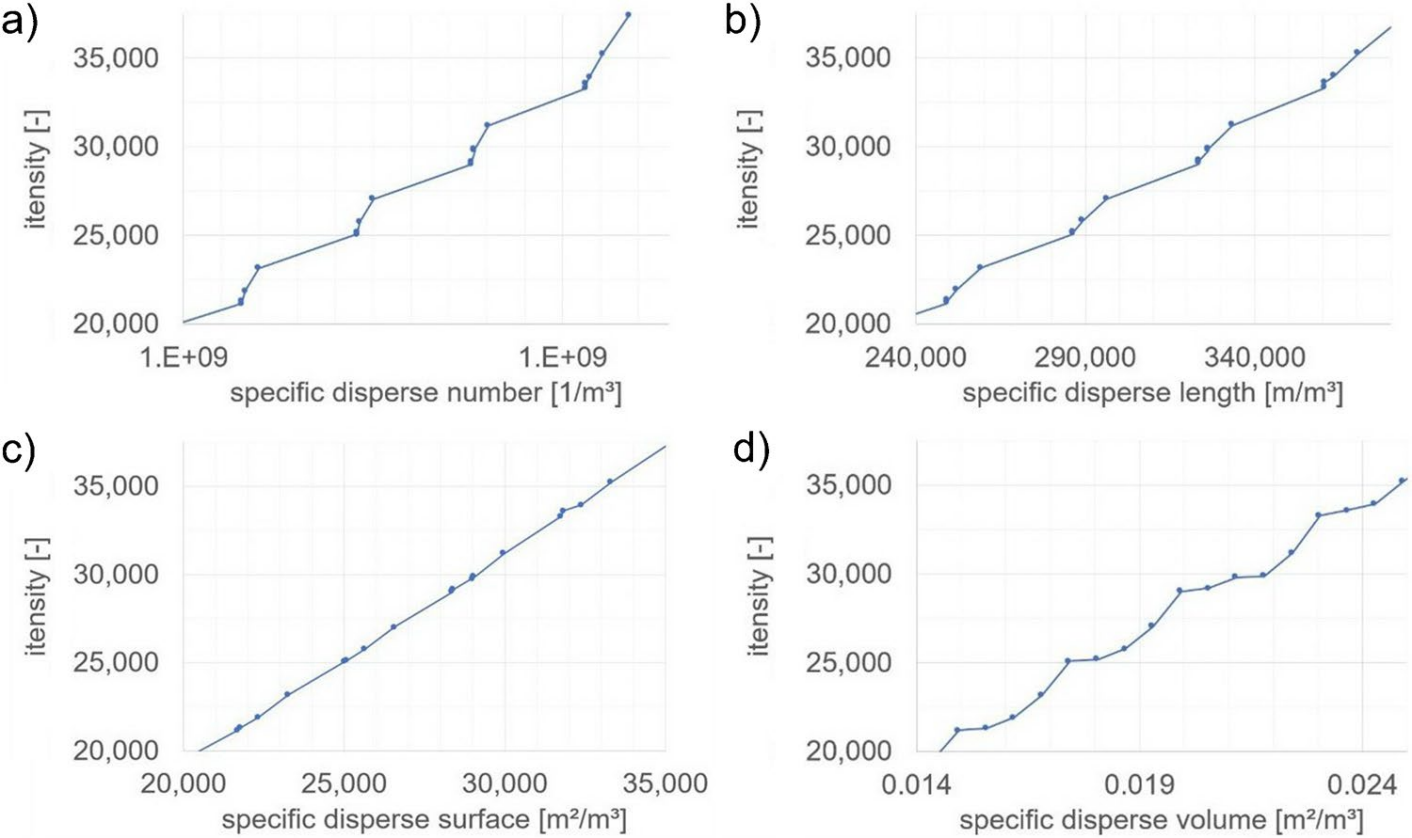
Close-up of sensor signal in the range of the intensity between 20,000 and 37,000 of dispersed glass beads over **a** specific dispersed number; **b** specific dispersed length; **c** specific dispersed surface; **d** specific dispersed volume

To determine the system parameters relevant for the signal intensity, the dispersed number (~ d32), the dispersed length (~ d¢32), the dispersed surface (~ d232), and the dispersed volume per volume (~ d332) of water were calculated from the weighed masses of the spheres and their Sauter diameters. This resulted in specific dispersed surface ranging from zero to approximately 71,000 m^2^m^−3^. For the graphical evaluations in Fig. 7, an average value was calculated over the last 2 min of each addition. A second-degree polynomial trend line was fitted across the data space no. II, and the coefficient of determination was determined. Within this interval, the curvature of the polynomial is low, resulting in a nearly linear appearance, as illustrated in the magnified view in Fig. 8c. Only data space no. II was used for the evaluation. It was not possible to collect enough data in data space no. I to draw any meaningful conclusions. Further research is required for an in-depth analysis of this area.

As shown in the paper 10.1002/cite.202200076 Schmitt et al. [1] on monodisperse suspensions of glass particles in clear aqueous solution, concentration series are also investigated here. This time, the volume concentration of large and small particles is varied alternately. As shown in Fig. 7c, the specific dispersed surface exhibits the strongest correlation with the measured signal among all investigated system parameters, with a coefficient of determination of R^2^ = 0.9996.

The scattered light signal is displayed in comparison to different characteristic values of the suspension. If the signal is plotted against the volume fraction (Fig. 8d), it can be seen that the addition of different particle fractions leads to varying slopes in the signal. However, this effect only becomes clearly visible when the measurement data is viewed in an enlarged section. For this reason, a close-up of the relevant range between 20,000 and 35,000 counts was visualized. The same is also true when the signal is plotted against the zero or first moment of the size distribution. The mixed signal appears to correlate poorly with the respective moment of the distribution.

The best correlation is obtained when all mixtures are plotted against the specific surface area (Fig. 8c). Even if slight systematic deviations are recognizable here, the correlation is significantly better than with the zero, first, or third moment of the distribution. The recognizable slight systematic deviations can even be explained by the different parameters according to the Mie simulation. Conversely, however, it is not possible to infer the particle size distribution backwards with only one given signal. However, the theoretical Mie approach offers initial approaches to this, which will be investigated in subsequent projects.

In addition to confirming the measurability of the disperse surface, the entire investigation also shows the following facts. The addition of fine material massively increases the dispersed surface. In a chemical reaction vessel with a limited surface area, this means that the small particles strongly dominate the chemical reaction that may be taking place. In relation to crystallization experiments, this also documents the risk of fine salt showers. The occurrence of fine salt showers results in an abruptly massively increased surface area, which strongly interferes with the reaction process. The results presented here therefore show the transferability of the measurement possibility of disperse surfaces with simple scattered light sensors to particle size distributions, very broad particle size distributions, and multimodal distributions even in the extreme range from 2 µm to 100 µm.

## Summary

Overall, this paper has shown that backscattering sensors can be used to directly measure the dispersed surface in suspensions, and that this statement also applies in particular to mixtures of large and small particles that are present in a common suspension. The particles used in this article range from 2 μm to 100 μm in diameter. Technologically, this finding is important in crystallizers, for example, where fine salt showers can occur. The small particles make a much larger contribution to the specific surface area than coarse particles. This can also be seen in the model concentration series carried out here and their measurement. The change in the concentration of the large particles only slightly increases the dispersed surface. The change in the fine particles results in the main impact here. The general statement that such a measurement can be carried out in a very high concentration range using a single scattered light signal is also important for technological applications. However, it should be borne in mind that this type of measurement with a single sensor cannot initially provide any information about the volume or mass concentration, or only if further information about the substance is also available. The question should now be asked whether a combination of sensors can achieve a statement that goes beyond the specific surface area. This is to be investigated in future work.

## Data Availability

The data supporting the findings of this study are available from the corresponding author upon reasonable request.

## Abbreviations

ATR: Attenuated reflection
FBRM: Focused beam reflectance measurement
PSD: Particle size distribution
QR: Quasi backscattering
QRW: Quasi backscattering with wide spacing
R^2^: Coefficient of determination
REM: Remission
SNR: Signal to noise ratio
SSC: Supersaturation control
TR: Transmission
Wt. %: Weight percent
